# Structural features and kinetic characterization of alanine racemase from *Staphylococcus aureus* (Mu50)

**DOI:** 10.1107/S0907444911050682

**Published:** 2011-12-09

**Authors:** Emma R. Scaletti, Sylvia R. Luckner, Kurt L. Krause

**Affiliations:** a Department of Biochemistry, University of Otago, Dunedin, New Zealand

**Keywords:** alanine racemase, *Staphylococcus aureus*

## Abstract

*Staphylococcus aureus* is an opportunistic Gram-positive bacterium which causes a wide variety of diseases ranging from minor skin infections to potentially fatal conditions such as pneumonia, meningitis and septicaemia. The pathogen is a leading cause of nosocomial acquired infections, a problem that is exacerbated by the existence of methicillin- and glycopeptide antibiotic-resistant strains which can be challenging to treat. Alanine racemase (Alr) is a pyridoxal-5′-phosphate-dependent enzyme which catalyzes reversible racemization between enantiomers of alanine. As d-alanine is an essential component of the bacterial cell-wall peptidoglycan, inhibition of Alr is lethal to prokaryotes. Additionally, while ubiquitous amongst bacteria, this enzyme is absent in humans and most eukaryotes, making it an excellent antibiotic drug target. The crystal structure of *S. aureus* alanine racemase (Alr*_Sas_*), the sequence of which corresponds to that from the highly antibiotic-resistant Mu50 strain, has been solved to 2.15 Å resolution. Comparison of the Alr*_Sas_* structure with those of various alanine racemases demonstrates a conserved overall fold, with the enzyme sharing most similarity to those from other Gram-positive bacteria. Structural examination indicates that the active-site binding pocket, dimer interface and active-site entryway of the enzyme are potential targets for structure-aided inhibitor design. Kinetic constants were calculated in this study and are reported here. The potential for a disulfide bond in this structure is noted. This structural and biochemical information provides a template for future structure-based drug-development efforts targeting Alr*_Sas_*.

## Abstract 2

The tertiary structure and kinetic properties of alanine racemase from *Staphylococcus aureus* are described and compared to other related alanine racemase structures.

## 1. Introduction

*Staphylococcus aureus* is a highly pathogenic Gram-positive coccus which was first discovered in the pus of surgical abscesses by Sir Alexander Ogston in 1883 (Ogston, 1883 ▶). *S. aureus* frequently colonizes the skin and has a niche preference for the anterior nares of the nose (Kluytmans *et al.*, 1997 ▶), with persistent nasal carriage occurring in 25–30% of the population (Gorwitz *et al.*, 2008 ▶). *S. aureus* can cause a wide variety of diseases ranging from skin infections such as impetigo and folliculitis to life-threatening diseases such as severe haemorrhagic pneumonia, meningitis, toxic shock syndrome and septicaemia (von Rittershain, 1878 ▶; Shands *et al.*, 1980 ▶; Lowry, 1998 ▶; Lina *et al.*, 1999 ▶). Host conditions which increase the likelihood of a severe *S. aureus* infection include factors such as open wounds, immunosupression and surgery (Laupland *et al.*, 2003 ▶; Giacometti *et al.*, 2000 ▶). *S. aureus* is a common cause of hospital-acquired infection (Lowry, 1998 ▶; Fluit *et al.*, 2001 ▶; Brumfitt & Hamilton-Miller, 1989 ▶), a problem that is exacerbated by the alarming rate at which this bacterium has developed antibiotic resistance.

Of particular concern is the rise in community-acquired methicillin-resistant *S. aureus* (MRSA), which has broad resistance to β-lactam antibiotics, including penicillins and cephalosporins. In the United States, MRSA is responsible for nearly 100 000 invasive infections and 19 000 deaths per year (Klevens *et al.*, 2007 ▶). MRSA was first reported in the United Kingdom in 1961 (Jevons, 1961 ▶) and has since become a problem in hospitals worldwide, heavily increasing dependence on vancomycin for treatment (Finland, 1979 ▶; Jernigan *et al.*, 1995 ▶; Weinstein & Fridkin, 2001 ▶). Further complicating this problem is the emergence of MRSA strains such as Mu50, which are also resistant to vancomycin. The first case of MRSA with intermediate resistance to vancomycin (VISA) was reported in 1996 (Hiramatsu *et al.*, 1997 ▶) and the first example of complete resistance to vancomycin (VRSA) was published in 2003 (Chang *et al.*, 2003 ▶). There are limited alternatives for treating VRSA infections, such as linezolid and daptomycin; however, these antibiotics can have serious side effects with prolonged use (Lin *et al.*, 2006 ▶; French, 2003 ▶; Echevarria *et al.*, 2005 ▶) and resistance to both has been observed (Skiest, 2006 ▶; Hayden *et al.*, 2005 ▶; Tsiodras *et al.*, 2001 ▶; Wilson *et al.*, 2003 ▶). This emphasizes the importance of identifying new drug targets for the future development of antibiotics which could be used to treat antibiotic-resistant *S. aureus* infections.

Alanine racemase (EC 5.1.1.1) is a pyridoxal-5′-phosphate (PLP) dependent enzyme which catalyzes the reversible racemization of l-alanine and d-alanine (Walsh, 1989 ▶). Alanine racemase is ubiquitous amongst bacteria but is absent in humans and rare in eukaryotes, with exceptions that include the fungi *Tolypocladium niveum* and *Cochliobolus carbonum* (Hoffmann *et al.*, 1994 ▶; Cheng & Walton, 2000 ▶), the yeast *Schizosaccharomyces pombe* (Uo *et al.*, 2001 ▶), bivalve molluscs (Matsushima & Hayashi, 1992 ▶; Nomura *et al.*, 2001 ▶) and some crustaceans (Shibata *et al.*, 2000 ▶; Fujita *et al.*, 1997 ▶; Yoshikawa *et al.*, 2002 ▶). As d-alanine is an essential component of the bacterial cell-wall peptidoglycan, inhibition of alanine racemase is lethal to those prokaryotic organisms that are dependent on this enzyme for d-alanine, thus making the enzyme an attractive antibiotic drug target (Lambert & Neuhaus, 1972 ▶). There are two alanine racemase isozymes in *S. aureus*: an anabolic alanine racemase (Alr) that is constitutively expressed at low levels and a catabolic alanine racemase (DadX) which is inducible by l-alanine. Gram-negative bacteria commonly possess both isozymes, in contrast to Gram-positive bacteria, which usually only possess the anabolic form of the enzyme (Wasserman *et al.*, 1983 ▶).

Structural studies of alanine racemase from bacteria such as *Geobacillus stearothermophilus*, *Pseudomonas aeruginosa*, *Streptococcus lavendulae*, *Mycobacterium tuberculosis*, *Escherichia coli*, *Bacillus anthracis* and *Enterococcus faecalis* indicate that the enzyme is a homodimer in its native con­formation (Shaw *et al.*, 1997 ▶; LeMagueres *et al.*, 2003 ▶, 2005 ▶; Noda *et al.*, 2004 ▶; Wu *et al.*, 2008 ▶; Au *et al.*, 2008 ▶; Couñago *et al.*, 2009 ▶; Priyadarshi *et al.*, 2009 ▶). Each monomer has two domains: an α/β-barrel at the N-terminus and a C-terminal domain which is predominantly β-stranded. The enzyme has two active sites, which are formed by interactions between the N-terminal domain of one monomer and the C-terminal domain of its partner. In both active sites the essential PLP cofactor resides in the mouth of the α/β-barrel, forming an *N*′-­pyridoxyl-lysine-5′-monophosphate (LLP) residue resulting from an internal aldimine linkage between PLP and a highly conserved catalytic lysine.

Numerous kinetic studies of *G. stearothermophilus* alanine racemase support the utilization of a stepwise two-base mechanism by the enzyme, in which a highly conserved active-site lysine and tyrosine act as general acid/base catalysts. These residues assume the role of either donating or abstracting an α-hydrogen from the substrate, depending on the direction in which the reaction is proceeding (Watanabe *et al.*, 1999 ▶, 2002 ▶; Sun & Toney, 1999 ▶; Spies & Toney, 2003 ▶).

Compounds shown to inhibit alanine racemase include the natural antibiotics d-cycloserine (DCS) and *O*-carbamoyl-d-serine (Neuhaus, 1967 ▶) and alanine analogues such as alanine phosphonate (Copié *et al.*, 1988 ▶), β-fluoroalanine, β-chloro­alanine (Wang & Walsh, 1978 ▶) and β,β,β-trifluoroalanine (Faraci & Walsh, 1989 ▶). Structural studies of alanine racemase with DCS bound indicate that its inhibition results from the formation of a stable covalent adduct between the antibiotic and PLP (Fenn *et al.*, 2003 ▶).

DCS is marketed for the treatment of *M. tuberculosis* infection; however, it has limited use as it can cause severe central nervous system toxicity (Newton, 1975 ▶; Yew *et al.*, 1993 ▶), which appears to arise from the inhibition of human enzymes that utilize PLP as a cofactor (Lambert & Neuhaus, 1972 ▶; Wang & Walsh, 1978 ▶). Other inhibitors which are not used clinically such as alanine phosphonate and propionate also target PLP and thereby suffer from the same lack of specificity (Stamper *et al.*, 1998 ▶; Morollo *et al.*, 1999 ▶). This emphasizes the need for the development of new inhibitors for alanine racemase with greater specificity, which may translate into less toxicity to humans.

Here, we report the successful purification, crystallization, structure determination and kinetic characterization of *S. aureus* alanine racemase from the Mu50 strain (Alr*_Sas_*), which exhibits resistance to both methicillin and glycopeptide antibiotics. Elucidation of the crystal structure of this enzyme is an important prerequisite for future structure-based drug-design efforts targeting *S. aureus* Alr.

## 2. Methods

### 2.1. Protein overexpression and purification

*E. coli* BL21 (DE3) cells were transformed with pMB1978 (obtained from Professor Michael Benedik, Texas A&M University), which contained the Alr*_Sas_* gene inserted into a pET26b background. Cells grown overnight at 310 K were used to inoculate the main culture, which was induced with IPTG at an OD_600_ of 0.5. Following expression for 14 h, the cell pellet was collected by centrifugation and the cells were lysed *via* sonication. After ammonium sulfate cuts of 30 and 70%, the resuspended pellet containing Alr*_Sas_* was dialysed against 20 m*M* Tris–HCl pH 7.6 and further purified by anion-exchange, hydrophobic interaction and finally size-exclusion chromatography. Peak fractions with greater than 95% purity as shown by SDS–PAGE were pooled and dialysed against 20 m*M* Tris–HCl pH 7.6 before crystallization.

### 2.2. Crystallization

Purified Alr*_Sas_* was concentrated to 12 mg ml^−1^ using a Vivaspin 2 concentrator (10 000 Da molecular-weight cutoff; GE Healthcare) and sitting drops were set up *versus* 100 m*M* sodium acetate trihydrate pH 5.0 and 1.8 *M* ammonium sulfate. Deep yellow crystals of 0.5 × 0.2 × 0.1 mm in size grew within 7 d and were cryoprotected by soaking them in mother liquor containing stepwise increasing amounts of glycerol from 5 to 30%.

### 2.3. Data collection and processing

A native Alr*_Sas_* data set was collected at 110 K on a Rigaku MicroMax-007 HF X-ray generator equipped with a rotating copper anode and Rigaku R-AXIS IV^++^ detector (Rigaku, Japan), using an oscillation angle of 0.5° and an exposure time of 5 min per image. Diffraction images were processed with *iMOSLFLM* (Battye *et al.*, 2011 ▶), *POINTLESS* (Grosse-Kunstleve *et al.*, 2002 ▶) and *SCALA* (Evans, 2006 ▶) within the *CCP*4 suite (Winn *et al.*, 2011 ▶). The crystals of Alr*_Sas_* belonged to the orthorhombic space group *P*2_1_2_1_2_1_ and diffracted to 2.15 Å resolution. Their unit-cell parameters were *a* = 65.1, *b* = 113.9, *c* = 126.0 Å, α = β = γ = 90°. Data-collection and processing statistics are presented in Table 1 ▶.

### 2.4. Structure determination and refinement

The structure of Alr*_Sas_* was solved *via* molecular replacement with *Phaser* (McCoy *et al.*, 2007 ▶) using the monomer of *G. stearothermophilus* Alr (with the PLP cofactor and waters removed), to which it has a sequence identity of 44%, as the search model. This was performed assuming two monomers per asymmetric unit, as suggested by the resulting Matthews coefficient *V*_M_ of 2.78 Å^3^ Da^−1^ (Matthews, 1968 ▶). *PHENIX* v.1.6.1 (Adams *et al.*, 2010 ▶) was used to build the initial model. After several rounds of manual model building using *Coot* (Emsley & Cowtan, 2004 ▶) and refinement using *REFMAC*5 (Murshudov *et al.*, 2011 ▶), the electron density improved and waters, the cofactor *N*′-pyridoxyl-lysine-5′-monophosphate, acetate and sulfate molecules were incorporated into the structure. The final structure had an *R* factor of 18.9% and an *R*_free_ value of 23.7%. Intermonomer interactions were analysed using the *Protein Interfaces, Surfaces and Assemblies* service (*PISA*) at the European Bioinformatics Institute (http://www.ebi.ac.uk/pdbe/prot_int/pistart.html; Krissinel & Henrick, 2007 ▶). The final model was validated using *PRO­CHECK* (Laskowski *et al.*, 1993 ▶), with the resulting Ramachandran plot indicating that 90.4% of the residues are in the most favoured regions, with the remaining 9.6% in additionally allowed regions. Additional structure-determination and refinement statistics are presented in Table 1 ▶.

### 2.5. Enzyme kinetics

The kinetic parameters *K*_m_ and *V*_max_ were determined using a spectrophotometric assay based on Esaki & Walsh (1986 ▶) and as described in our previous work (Strych *et al.*, 2000 ▶, 2001 ▶). Substrate concentrations of l- and d-alanine (10, 5, 2.5, 1.6 and 1.0 m*M*) were assayed in triplicate using 20 ng Alr*_Sas_* per reaction. The consumption or production of NADH (340 nm) was monitored for 10 min at 303 K, after which the kinetic constants *K*_m_ and *V*_max_ were determined using linear regression to a Lineweaver–Burk model as well as using nonlinear regression fitting carried out within *GraphPad Prism* v.5 (GraphPad Software, La Jolla, California, USA).

### 2.6. Dynamic light scattering

Dynamic light scattering (DLS) was performed using a DynaPro-99 system (Wyatt Technologies). Purified Alr*_Sas_* (1 mg ml^−1^) was filtered with a 0.02 µm Anotop filter (Whatman) and added to a quartz light-scattering cuvette at 293 K. *DYNAMICS* software was used to calculate the radius of hydration, molecular weight and percent polydispersity.

### 2.7. Mass spectrometry

To prepare the sample for MALDI–TOF analysis, crystals of Alr*_Sas_* were washed, crushed, dissolved in 20 m*M* Tris–HCl pH 7.5 and run on an SDS–PAGE gel. The Alr*_Sas_* band was excised and digested with trypsin, after which a solution consisting of 30% acetonitrile and 0.1% trifluoroacetic acid was added to the samples. The digested solution was mixed with a matrix solution comprised of 10 mg ml^−1^ α-cyano-4-­hydroxycinnamic acid dissolved in 65% acetonitrile (containing 0.1% trifluoroacetic acid and 10 m*M* ammonium dihydrogen phosphate). This mixture was applied onto a MALDI sample plate and air-dried. MS analysis of the samples was performed using a 4800 MALDI tandem time-of-­flight analyzer (MALDI–TOF/TOF). For disulfide-bond analysis a 1 mg ml^−1^ sample of Alr*_Sas_* in solution was prepared as above, with the addition of an additional step prior to tryptic digestion in which the sample was alkylated with 20 m*M* iodacetamide. The resulting sample was analyzed using an LTQ-Orbitrap (MS/MS) instrument coupled to a nanoflow liquid-chromatography system.

## 3. Results and discussion

### 3.1. Overall structure of *S. aureus* alanine racemase

**Figure 1:**
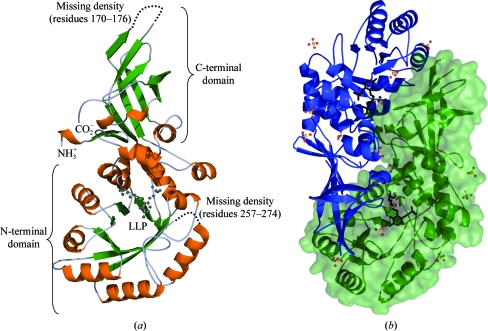
(*a*) Structure of the *S. aureus* alanine racemase monomer. Ribbon representation with α-helices coloured orange and β-sheets shown in green. The PLP cofactor covalently bound to Lys39 is shown as a black stick model. (*b*) Ribbon representation of the *S. aureus* alanine racemase dimer. Monomers are coloured blue and green, with the surface representation of one monomer also shown in green. The PLP cofactors are depicted as black stick models. Sulfate and acetate molecules are shown as ball-and-stick models; C atoms are coloured black, O atoms red, S atoms yellow and phosphates orange.

The tertiary structure of *S. aureus* alanine racemase (Alr*_Sas_*) is a homodimer comprised of two identical monomers (Fig. 1 ▶), consistent with previous crystallographic studies of alanine racemases (Shaw *et al.*, 1997 ▶; LeMagueres *et al.*, 2003 ▶, 2005 ▶; Wu *et al.*, 2008 ▶; Couñago *et al.*, 2009 ▶). However, despite numerous structural studies having indicated that the enzyme is dimeric, there have been reports of some alanine racemases having a different tertiary structure in solution. In particular, the alanine racemases from *T. niveum* and *Corbicula japonica* have been suggested to be either trimeric or tetrameric in solution (Hoffmann *et al.*, 1994 ▶). In order to determine the oligomeric state of Alr*_Sas_* in solution, we performed dynamic light scattering (DLS), which yielded a monodisperse single peak account­ing for 98.6% of the sample mass, with a radius of hydration of 3.7 nm and a calculated molecular mass of 73 kDa. As the hypothetical molecular weight of the Alr*_Sas_* monomer is 42.8 kDa, this result is most consistent with the enzyme being predominantly dimeric in solution.

Inspection of our X-ray structure revealed that the Alr*_Sas_* homodimer has two active sites, both of which are comprised of residues from the N-terminal domain of one monomer and residues from the C-terminal domain of the second monomer. In this way, both monomers contribute to the entryway and active site of the enzyme (Fig. 1 ▶ *b*). Each Alr*_Sas_* monomer is comprised of two distinct domains. The N-terminal domain corresponds to residues 1–241 in the structure and consists of an eight-stranded α/β-barrel. The essential PLP cofactor is covalently bound to the highly conserved catalytic lysine (Lys39) *via* an internal aldimine linkage and extends towards the centre of the α/β-barrel. The C-terminal domain consists of residues 242–382 and has a secondary structure that is predominantly β-stranded. This region contains three antiparallel β-sheets, with the exception of one β-sheet in which two out of the five β-strands are parallel (Fig. 1 ▶ *a*). The individual Alr*_Sas_* monomers are crystallographically distinct and form a dimer in the asymmetric unit. Following refinement they have a low r.m.s difference of 0.41 after C^α^-atom superposition.

**Figure 2:**
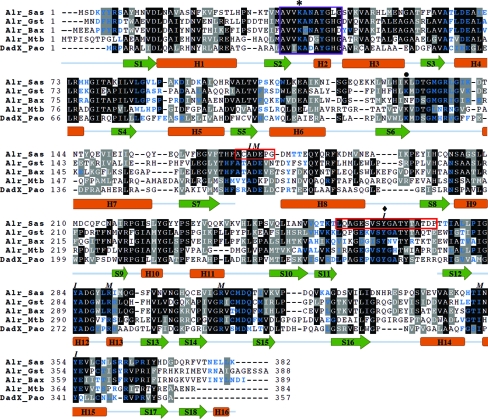
Structure-based sequence alignment of alanine racemases from *S. aureus* (Alr_Sas), *G. stearothermophilus* (Alr_Gst), *B. anthracis* (Alr_Bax), *M. tuberculosis* (Alr_Mtb) and *P. aeuruginosa* (DadX_Pao). Identical residues are shaded black, while grey shading indicates amino acids with conserved physicochemical properties. The purple box encloses the conserved PLP-binding motif and the red boxes correspond to missing density in the Alr*_Sas_* structure. *I* and *M* represent residues that form the inner and middle layers of the active-site entryway. The asterisk marks the highly conserved PLP-bound lysine, the diamond marks the location of the catalytic tyrosine and the bullet point indicates the location of a residue which is often carbamylated in alanine racemases which have a lysine at this position. The secondary structure corresponding to the amino-acid sequence of Alr*_Sas_* is shown, with α-helices coloured orange and β-strands coloured green. Residues involved in the dimer interface are shown as blue letters.

The Alr*_Sas_* structure is lacking clear density for residues 170–176 and 257–274 in both monomers and these regions are indicated by red boxes in Fig. 2 ▶. Important amino acids in these missing areas include Ala171, Asp173 and the highly conserved catalytic tyrosine Tyr265′. These residues are known from previous structural studies to contribute to the active-site entryway. The missing density in the N-terminus (170–176) in other known alanine racemase structures corresponds to a small loop in the α/β-barrel between β-strand 7 and helix 8, whereas the C-terminal region (257–274) usually contains additional β-structure between β-­strands 11 and 12 (Shaw *et al.*, 1997 ▶; LeMagueres *et al.*, 2003 ▶, 2005 ▶; Couñago *et al.*, 2009 ▶). As the overall monomer topology is very similar between Alr*_Sas_* and the other enzymes, it is likely that Alr*_Sas_* would also have the same secondary structure in these areas. Similar regions were missing in the structure reported for Alr*_Mtb_*, but the missing regions were not observed in both monomers. Notably, mass-spectrometric analysis of samples from crushed Alr*_Sas_* crystals confirmed the presence of both regions that are missing density in the crystal structure.

Two acetate molecules and 13 sulfates were modelled into the Alr*_Sas_* structure. This observation was not surprising as sodium acetate trihydrate and ammonium sulfate were present in the crystallization conditions. Analysis of the positions of these sulfates showed that all but one is found in the same position in both monomers and they maintain the same non­covalent interactions. Eight sulfates are located in solvent-exposed areas, whereas the remaining four are found within the dimer interface of Alr*_Sas_*. One sulfate is located on a point of symmetry in the dimer interface and interacts with Arg362 and Ser361 from both monomers. Sulfate molecules are also a feature of the alanine racemase structure from *E. coli* (Wu *et al.*, 2008 ▶), but there are only three sulfates per monomer, none of which are in the same location in these two structures.

During the early stages of the refinement, we noted weak positive density between Cys201 and Cys215 in each monomer. As the refinement progressed the intra-sulfur density diminished and the residues were built as cysteine not cystine. The final refined electron density and geometry are most consistent with cysteine, but we do note an intra-sulfur distance of 3.2 Å, which is less than the sum of the van der Waals radii of the two sulfurs. To help address the disulfide question, we investigated alkylated and un­alkylated digests of Alr*_Sas_* using mass spectrometry (data not shown). The results of this study were more supportive of cysteines at these positions, but could not rule out some degree of disulfide formation.

Disulfide-bond formation has not been observed at this, or any other, position in any alanine racemase studied to date. There is no suggestion that this bond would have any role in catalysis or regulation. Furthermore, these two cysteines are not conserved in other alanine racemases, which effectively rules out an important role for a disulfide at this position. We note that there is an unpublished *S. aureus* Alr structure solved to 2.37 Å resolution in the Protein Data Bank (PDB) which was also crystallized at pH 5.0 (PDB entry 3oo2; Center for Structural Genomics of Infectious Diseases, unpublished work). In this structure a disulfide bond is reported between these two residues, but an account of this structure has not yet been published and no further information is available. In our view, the most consistent interpretation of the data described above is that the structure may contain a mixture of disulfide and dithiol forms depending on the local environment but that these two cysteines are found predominantly in their reduced form in the Alr*_Sas_* structure that we report here.

### 3.2. Structural and biochemical comparison with closely related alanine racemases

#### 3.2.1. Structural comparisons

Superposition of the C^α^ atoms from the Alr*_Sas_* monomer with the anabolic alanine racemases from *G. stearothermophilus* (Alr*_Gst_*), *B. anthracis* (Alr*_Bax_*) and *M. tuberculosis* (Alr*_Mtb_*), and the catabolic alanine racemase from *P. aeruginosa* (DadX*_Pao_*) indicates a high level of structural identity between the enzymes. As shown in Table 2 ▶, the Alr*_Sas_* monomer is most similar structurally to those of Alr*_Gst_* and Alr*_Bax_*, with which it has highest percent amino-acid sequence identity. The Alr*_Sas_* monomer shares somewhat less structural identity when compared with Alr*_Mtb_* and DadX*_Pao_*, as indicated by the higher r.m.s. differences and lower percent sequence identities. The structure-based sequence alignment presented in Fig. 2 ▶ indicates important residues that are conserved across these enzymes. Such con­served regions include the PLP-binding motif consisting of residues AVV­KANAYGHG, which is located at the beginning of the α/β-barrel domain. This motif contains the highly conserved catalytic lysine (Lys39) covalently bound to the essential PLP cofactor. The nonconserved differences in this sequence are Asn41, which is an aspartate in Alr*_Mtb_* and DadX*_Pao_*, Ala40 and Gly46, which are a glycine and an aspartate, respectively, in Alr*_Bax_*, and Leu45, which is a histidine in the other listed structures.

Comparison of the alanine racemase monomers indicates that they share very similar overall topology. C^α^-atom superpositions of the individual domains and active-site residues from Alr*_Sas_* with those of the other structures are presented in Table 2 ▶. As was the case with whole monomers, the individual domains of Alr*_Sas_* are the most similar structurally to those from the Gram-positive bacteria Alr*_Gst_* and Alr*_Bax_*, as indicated by the low r.m.s. differences for the N-terminal and C-­terminal regions. The individual domains of Alr*_Sas_* share less structural similarity with Alr*_Mtb_* and DadX*_Pao_*, having higher r.m.s. differences for both domains. In each comparison the C-­terminal domains were shown to consistently superpose better than the N-terminal domains, with both regions having lower r.m.s. differences compared with whole monomers. However, the active-site residues of all of the alanine racemases surveyed superpose particularly well with Alr*_Sas_*, with low r.m.s. differences. When compared with the higher r.m.s. differences observed for superpositions involving the whole monomers and individual domains of these alanine racemases, this demonstrates the ability of these enzymes to tolerate deviations between domains while still retaining very similar active sites (LeMagueres *et al.*, 2003 ▶).

#### 3.2.2. Structural deviations

**Figure 3:**
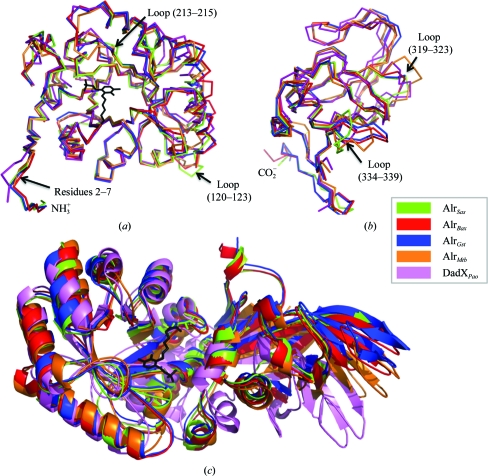
C^α^-atom superpositions of Alr*_Sas_* and other alanine racemases. *S. aureus*, green; *G. stearothermophilus*, blue; *B. anthracis*, red; *M. tuberculosis*, orange; *P. aeruginosa*, purple. C^α^-atom traces showing superpositions between the (*a*) N-terminal and (*b*) C-terminal domains. Regions corresponding to significant structural deviations and their location in the Alr*_Sas_* structure are labelled. (*c*) Superposition of the N-terminal α/β-barrel domain of whole alanine racemase monomers visualized as a ribbon representation. The PLP cofactor of Alr*_Sas_* is depicted as a black stick model.

The individual N-terminal and C-­terminal domains of Alr*_Sas_* and the other alanine racemases generally superpose well, but there are three notable examples of structural divergence between these enzymes (Fig. 3 ▶ *a*). The first example is near the N-terminus (residues 2–7), within which the Alr*_Sas_* structure deviates significantly from that of DadX*_Pao_* but superposes well with those of other alanine racemases. The reason for this difference could be attributed to the fact that DadX*_Pao_* has fewer residues in this area compared with the other enzymes. As illustrated in Fig. 2 ▶, this region contributes to the dimer interface in each of the structures. The second and third examples relate to two loop regions (residues 120–123 and 213–215) of the Alr*_Sas_* structure which deviate from each of the compared alanine racemases. *PDBePISA* analysis (Krissinel & Henrick, 2007 ▶) verifies that both of these loop regions are in solvent-exposed areas which do not contribute to the dimer interface of the protein (Fig. 2 ▶). The *B* factors for region 120–123 are 39 Å^2^ (main chain) and 45 Å^2^ (side chains), which are high compared with those of the overall structure of Alr*_Sas_* (main-chain atoms, 20.3 Å^2^; side-chain atoms, 22.5 Å^2^), suggesting that this area is flexible. In con­trast, the *B* factors for region 213–215 do not differ significantly from those of the overall structure (25 and 29 Å^2^ for the main chain and side chains, respectively).

In the C-terminal domain there are two main examples of structural divergence (Fig. 3 ▶ *b*). The first example is a small loop in Alr*_Sas_* (residues 319–323) which superimposes relatively well with the other alanine racemases with the exception of Alr*_Mtb_*. In Alr*_Mtb_* this solvent-exposed loop is three amino acids longer than the equivalent region in Alr*_Sas_*. The structural divergence in this region of Alr*_Mtb_* is likely to be the result of a more complicated secondary structure in this area, which is a combination of β-structure and loop (LeMagueres *et al.*, 2005 ▶) rather than purely loop as is observed for Alr*_Sas_*. The second example of structural divergence in the C-terminal domain is a solvent-exposed loop corresponding to residues 334–339 in the Alr*_Sas_* structure which diverges from each of the other alanine racemases. The *B* factors in this area of Alr*_Sas_* are 21.0 Å^2^ (main chain) and 22.9 Å^2^ (side chains), which are not high compared with those of the whole structure. There are no crystal contacts in this region and the density is of good quality. Overall, the areas of Alr*_Sas_* which show the greatest structural differences from the other alanine racemases are solvent-exposed loop regions predominantly located towards the ends of the individual domains, which are not involved in dimerization (with the exception of loop 2–7) and do not contribute to the active-site region of the enzyme. As these areas are generally less important in terms of enzymatic function, they may be more tolerant of such structural deviations.

#### 3.2.3. Hinge angle

Each enzyme monomer has a characteristic hinge angle between its N- and C-terminal domains, as presented in Fig. 3 ▶(*c*), which is the reason that individual monomers of the enzyme are unable to be optimally superimposed. Alr*_Sas_* has a hinge angle of 128.9°, which is intermediate between those of Alr*_Gst_* and Alr*_Bax_*, which have hinge angles of 129.4 and 127.6°, respectively. The interdomain angle has less similarity to that of Alr*_Mtb_* (130.9°) and differs most markedly from that of DadX*_Pao_* (133.9°), with which it consistently had the highest r.m.s. differences in terms of tertiary structure. The differences between these hinge angles are small in absolute terms, but the availability of two Alr*_Bax_* structures solved independently in different space groups showing the same hinge angle suggests that this feature of these enzymes is genuine (Au *et al.*, 2008 ▶; Couñago *et al.*, 2009 ▶). These distinct hinge angles are proposed to be mediated in part by hydrogen bonding between the N- and C-­terminal tails of opposite monomers (Le Magueres *et al.*, 2003 ▶). Longer alanine racemases such as Alr*_Sas_*, Alr*_Gst_* and Alr*_Bax_* that have extra residues in these regions are able to form these bonds, resulting in very similar hinge angles. Shorter alanine racemases such as Alr*_Mtb_* and DadX*_Pao_* lack the equivalent residues, particularly those found in the C-­terminus, and cannot form these bonds.

#### 3.2.4. Enzyme kinetics

Kinetic characterization of Alr*_Sas_* revealed a *V*_max_ of 250 U mg^−1^ and a *K*_m_ of 2.77 m*M* for the racemization of l-alanine to d-alanine and a *V*_max_ of 91 U mg^−1^ and a *K*_m_ of 0.89 m*M* for the opposite direction. It is noted that the activity of Alr*_Sas_* differs most markedly from that of Alr*_Gst_* (l→d *V*_max_ = 2550 U mg^−1^; d→l *V*_max_ = 1400 U mg^−1^), with which it shares the most similarity structurally (Table 3 ▶). Also, the l-alanine to d-alanine direction is kinetically favoured in Alr*_Sas_*, which makes evolutionary sense as this enzyme is the sole metabolic source of d-alanine, an essential cell-wall component. This is in agreement with the trend observed for Alr*_Gst_* and contrasts with Alr*_Mtb_* (l→d *V*_max_ = 0.51 U mg^−1^; d→l *V*_max_ = 0.46 U mg^−1^) and DadX*_Pao_* (l→d *V*_max_ = 155 U mg^−1^; d→l *V*_max_ = 134 U mg^−1^), in which neither direction of the racemization is favoured. Such inferences are unable to be drawn regarding Alr*_Bax_* as only the l-­alanine to d-alanine direction of the racemization has been characterized (*V*_max_ = 101 U mg^−1^). The range of values of *K*_eq_ calculated using the Haldane relationship for these enzymes is very close to one (between 0.89 and 1.16), as would be expected for a racemization reaction. Remarkably, despite high levels of sequence identity and very similar active-site structures, there can be up to three orders of magnitude difference in terms of their catalytic rates (Inagaki *et al.*, 1986 ▶; Strych *et al.*, 2000 ▶, 2001 ▶; Couñago *et al.*, 2009 ▶). Comparison of the hinge angle and kinetic constants of Alr*_Sas_* with those of the other alanine racemases shows that there is no relationship between these factors, but a recent publication involving seven different alanine racemases demonstrated a positive correlation between dimerization affinity and increased catalytic rate (Ju *et al.*, 2011 ▶).

#### 3.2.5. Dimer interface

Mutational studies of alanine racemases from *E. coli* and *P. aeruginosa* have demonstrated that dimerization is essential for enzyme activity (Strych & Benedik, 2002 ▶). The structure-based sequence alignment represented in Fig. 2 ▶ indicates that numerous residues which are involved in dimerization are highly conserved across the compared alanine racemases.

The area of the dimer interface of Alr*_Sas_* is 2510 Å^2^, which is smaller than those in Alr*_Gst_* (3083 Å^2^) and Alr*_Bax_* (3529 Å^2^) but larger compared with Alr*_Mtb_* and DadX*_Pao_* (1927 and 1917 Å^2^, respectively). The larger area of dimerization for Alr*_Gst_* and Alr*_Bax_* has previously been attributed to these enzymes having extra residues in the N- and C-termini involved in the interface which are not present in shorter enzymes such as DadX*_Pao_* (LeMagueres *et al.*, 2003 ▶; Couñago *et al.*, 2009 ▶). Fig. 2 ▶ indicates that these additional N- and C-­terminal residues are also present in the Alr*_Sas_* structure.

Calculation of the dimer interface of Alr*_Sas_* is problematic as it contains missing density in some regions of each monomer that are conserved and that contribute to the dimer interface in the other alanine racemases. To address this issue, we performed *PDBePISA* analysis (Krissinel & Henrick, 2007 ▶) taking the absent density from Alr*_Sas_* into account. This was achieved by modifying the PDB files of the other alanine racemases to remove the regions equivalent to those shown to be missing in the Alr*_Sas_* structure. When this change was included in the calculation of the dimer interfaces the result for Alr*_Sas_* (2510 Å^2^) was most comparable to that of Alr*_Gst_* (2579 Å^2^), with which it also shares the most structural similarity. The interface is still smaller compared with that in Alr*_Bax_* (2920 Å^2^) and larger than those in Alr*_Mtb_* and DadX*_Pao_* (1625 and 1614 Å^2^, respectively), albeit to a lesser degree in each case. Disruption of dimerization has been successfully employed as a strategy to inhibit protein drug targets in diseases such as HIV (Song *et al.*, 2001 ▶; Boggetto & Reboud-Ravaux, 2002 ▶). The absolute requirement of dimerization for alanine racemase function and the high level of conservation across these enzymes makes the dimer interface a possible target for structure-aided drug design.

### 3.3. Active site

**Figure 4:**
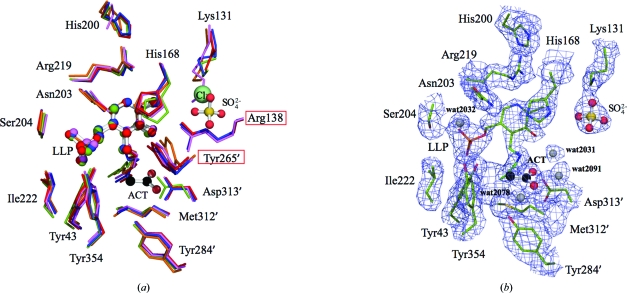
Active site of *S. aureus* alanine racemase. (*a*) Superposition of the active-site residues of alanine racemases from *S. aureus* (green), *G. stearothermophilus* (blue), *B. anthracis* (red), *M. tuberculosis* (orange) and *P. aeruginosa* (purple). The chloride present in the Alr*_Bax_* structure is shown as a pale green sphere. Residues labelled in red boxes lack density in the Alr*_Sas_* structure. (*b*) 2*F*_o_ − *F*_c_ electron-density map of the active site contoured at 1.0σ. The side chains of the Alr*_Sas_* active site are depicted as sticks; C atoms are coloured green, O atoms red, N atoms blue, S atoms yellow and phosphate orange. The PLP cofactors from each structure are depicted as ball-and-stick models. Important water molecules that are alluded to in the text are shown as grey spheres. In both panels the acetate and sulfate from the Alr*_Sas_* structure are represented as ball-and-stick models; C atoms are coloured black, O atoms red and S atoms yellow. Primed numbers denote residues that are contributed by the second monomer.

The active-site residues of Alr*_Sas_* and the other alanine racemases superpose particularly well with low r.m.s. differences, as indicated in Table 2 ▶ and Fig. 4 ▶(*a*). The active-site structure of Alr*_Sas_* is most similar to those of Alr*_Gst_* (r.m.s.d. 0.48 Å) and Alr*_Bax_* (r.m.s.d. 0.53 Å), with which it has the highest percent amino-acid sequence identity (70 and 68%, respectively). Alr*_Sas_* shares less similarity with Alr*_Mtb_* (r.m.s.d. 0.72 Å, 51% sequence identity) and diverges most from DadX*_Pao_* (r.m.s.d. 0.82 Å, 47% sequence identity), in accordance with the other C^α^-atom superpositions. As mentioned above, the enzyme active site is comprised of residues from both monomers, several of which are involved in the hydrogen-bond network of the PLP cofactor.

Fig. 4 ▶(*b*) depicts the active-site structure of Alr*_Sas_*, in which PLP is bound to the enzyme *via* an internal aldimine formed between the C4′ atom of the cofactor and Lys39 NZ. The internal aldimine is also within hydrogen-bonding distance of the phenolic O atom of PLP, which interacts with an ordered water molecule found in both active sites (wat2031 and wat2135). The cofactor exists in a dynamic equilibrium between the externally and internally reacted aldimine forms (LeMagueres *et al.*, 2003 ▶) and is capable of catalyzing reactions other than racemization, such as transamination and decarboxylation. As PLP alone is sufficient to catalyze these reactions, it has been suggested that the holoenzyme plays a critical role in controlling the reaction specificity of the co­factor (Toney, 2005 ▶). In alanine racemases the catalytic lysine and tyrosine are situated on opposite sides of the pyridine ring, which is proposed to aid in racemization being favoured over other PLP-dependent reactions (Shaw *et al.*, 1997 ▶). In the Alr*_Sas_* structure the phosphate tail of PLP is stabilized by interactions with residues from the first monomer. Of the three phosphate-group O atoms, OP_1_ hydrogen bonds to Ile222 NH and Tyr43 OH, OP_2_ interacts with Tyr354 OH and OP_3_ hydrogen bonds to Ser204 OH and NH. In addition, OP_3_ hydrogen bonds to a water molecule (wat2032) which also interacts with Ser204 NH and the side chain of Asn203.

The pyridine ring N1 of the PLP in Alr*_Sas_* is further stabilized *via* a hydrogen bond to the side chain of Arg219. This residue is in turn held in place by interactions with His200 and His168, the latter of which has been shown to interact with the phenolic O atom of Tyr265′ in other structures (Shaw *et al.*, 1997 ▶; LeMagueres *et al.*, 2003 ▶). Mutational studies of this arginine in Alr*_Gst_* have indicated that a positive charge at this position is essential for efficient catalysis owing to an electrostatic interaction with Tyr265′ *via* His168 which results in a lowering of the p*K_a_* of this important catalytic residue (Sun & Toney, 1999 ▶). In addition, Arg219 is also proposed to aid in the relative destabilization of the carbanionic intermediate by preventing protonation of the pyridine ring N1, thereby favouring racemization over other PLP-catalysed reactions (Spies & Toney, 2003 ▶).

Lys131 in Alr*_Sas_* is frequently carbamylated in alanine racemases and this was first reported in Alr*_Gst_* (Morollo *et al.*, 1999 ▶). It was proposed that a carbamylated lysine in this position would be important for the correct positioning and charge modulation of Arg138. In Alr*_Sas_* the density for Lys131 is not consistent with carbamylation and in addition there is poor density for the side chain of Arg138, implying that this residue may be poorly ordered. Notably, in place of a carbamylated lysine the Alr_Sas_ structure contains a bound sulfate molecule. This sulfate in Alr*_Sas_* interacts with the side chain of Lys131 and His200 and is within hydrogen-bonding distance of Arg138. A sulfate at this location has not been observed in the active sites of other alanine racemases, but reinforces the notion that a negative charge at this position is important for structural integrity in this region of the enzyme. Alanine racemases such as Alr*_Bax_* that lack a Lys129 equivalent have been shown in at least one case to utilize a chloride ion which may assume the same function as the carbamylated lysine (Couñago *et al.*, 2009 ▶). However, in the Alr*_Sas_* structure, while it looks as if this sulfate is substituting for a carbamate or chloride ion at this position, it is likely to be too bulky and highly charged to do so effectively, which may offer an explanation as to why Arg138 is poorly ordered.

It is likely that the absence of carbamylation of Lys131 in Alr*_Sas_* is a consequence of the low pH (5.0) at which the enzyme was crystallized, an effect which has been reported previously for the Class D OXA-10 β-lactamase (Golemi *et al.*, 2001 ▶). Alr*_Gst_* and DadX*_Pao_* (Morollo *et al.*, 1999 ▶; LeMagueres *et al.*, 2003 ▶), both of which demonstrate carbamylation of the equivalent lysine residue, were crystallized at a much higher pH than Alr*_Sas_* (8.5 and 7.5, respectively). It could not be established whether Alr*_Mtb_*, which was crystallized at pH 9.2, is carbamylated because of poor electron density for the side chain of the lysine residue corresponding to this position (LeMagueres *et al.*, 2005 ▶).

In the structure of Alr*_Sas_* there is one acetate, which is a weak inhibitor of alanine racemase, per active site. The carboxylate group of the Alr*_Sas_* acetate forms a hydrogen bond to Met312′ NH, analogous to the interactions made by the carboxylate group of alanine (Watanabe *et al.*, 2002 ▶). In addition, the carboxylate bound at this location also interacts with ordered water molecules in each active site (wat2091 and wat2078). These acetates are both bound near to the established substrate-binding site where other negatively charged inhibitors such as 1-aminoethylphosphonic acid (l-Ala-P) and propionate have also been observed (Stamper *et al.*, 1998 ▶; Morollo *et al.*, 1999 ▶). Acetate is a feature of the active sites of Alr*_Gst_* and Alr*_Bax_*, both of which require acetate for crystallization (Shaw *et al.*, 1997 ▶; Couñago *et al.*, 2009 ▶). In these structures acetate also hydrogen bonds to the guanidine group of Arg138 and the phenolic O atom of Tyr265′, but these interactions cannot be reported for Alr*_Sas_* owing to the absence of density for these two side chains. A consensus acetate-binding site for alanine racemase can be constructed by superposing the active sites from the four structures that contain either bound acetate or propionate and then averaging the locations of the acetate ligands. Analysis of this consensus acetate site indicates that it is well conserved, with r.m.s. deviations from this site of 0.43 Å for Alr*_Sas_*, 0.66 Å for Alr*_Gst_*, 0.74 Å for Alr*_Gst_* propionate and finally 0.9 Å for Alr*_Bax_*.

### 3.4. Active-site entryway

In agreement with previous structural analyses, the active-site entryway of Alr*_Sas_* is comprised of residues from both monomers and is roughly conical in shape, with the base of the cone oriented towards the outside of the enzyme. The narrow entryway consists of outer, middle and inner layers, which become increasingly constricted leading towards the PLP cofactor. As demonstrated in Fig. 2 ▶, the residues contributing to the inner (Ala172, Tyr265′, Tyr284′ and Tyr354) and middle (Asp173, Arg290′, Arg309 and Ile352) layers of the Alr*_Sas_* entryway are highly conserved between the compared alanine racemases. The residues which form the outer layer of the active-site entryway are not highly conserved between these enzymes (LeMagueres *et al.*, 2005 ▶). While the active-site binding pockets of alanine racemases are large enough to bind inhibitors, the constricted nature of the entryway could make it problematic for larger compounds to gain access to the active site. Structural studies of DadX*_Pao_* indicate that the greatest restriction in the entryway is between two tyrosines from the inner layer (Tyr253′ and Tyr341, DadX*_Pao_* num­bering), which are around 2.7 Å apart (LeMagueres *et al.*, 2003 ▶). At such a narrow distance one or both of these residues must move in order to permit even small substrates, namely enantiomers of alanine, to enter and leave the active site. Proteolytic digestion experiments in *S. typhimurium* (Galakatos & Walsh, 1987 ▶) suggest that the catalytic tyrosine (Tyr265′ in Alr*_Sas_*) is part of a conserved flexible loop in the entryway of the active site and mutational studies in *G. stearothermophilus* (Patrick *et al.*, 2002 ▶) suggest that the juxtaposed tyrosine (Tyr354 in Alr*_Sas_*) also plays a role in controlling substrate specificity. It may be possible, however, to direct drug-design efforts at residues that form the conserved portion of the entryway but do not need to extend beyond these two gating tyrosines (Im *et al.*, 2011 ▶).

The conserved active-site entryway, dimer interface and active-site binding pocket of alanine racemase are possible targets for structure-aided drug design. In each of these areas, the targeting of multiple residues as opposed to a single site (such as the enzyme cofactor) could be advantageous for the development of drugs with greater specificity for alanine racemases. The structural determination and biochemical characterization of Alr*_Sas_* presented here will provide a template for future structure-based drug-design studies of the enzyme, with the ultimate goal of developing new treatments for antibiotic-resistant strains of *S. aureus*.

## Supplementary Material

PDB reference: alanine racemase, 4a3q

## Figures and Tables

**Table 1:** Data-collection and refinement statistics Values in parentheses are for the highest resolution shell.

Space group	*P*2_1_2_1_2_1_
Unit-cell parameters	
*a* (Å)	65.1
*b* (Å)	113.9
*c* (Å)	126.0
α = β = γ (°)	90
Observations	187093 (24483)
Unique reflections	49388 (6693)
Completeness (%)	96.1 (91.0)
*R*_merge_† (%)	5.8 (19.4)
〈*I*/σ(*I*)〉	15.2 (6.3)
Multiplicity	3.8 (3.7)
Resolution range (Å)	51.91–2.15 (2.27–2.15)
*R* factor‡ (%)	18.9
*R*_free_ (%)	23.7
Average *B* factors (Å^2^)	
Wilson *B* factor	26.7
Main chain	20.3
Side chains	22.5
Waters	22.6
R.m.s. deviations	
Bond lengths (Å)	0.014
Bond angles (°)	1.35
No. of residues	
Protein atoms	5644
PLP atoms	30
Acetate atoms	8
Sulfate atoms	65
Water atoms	266

† *R*_merge_ = 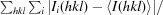 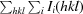.

‡ *R* = 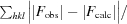 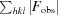.

**Table 2:** Average r.m.s. differences (Å) between the C^α^ atoms of Alr*_Sas_* and other alanine racemases The numbers in parentheses denote sequence identity with Alr*_Sas_*. Residues from the other structures equivalent to those in Alr*_Sas_* were used for the superpositions.

Alanine racemase	PDB code	Whole monomers†	N-terminal domain‡	C-terminal domain§	Active site¶
Alr*_Gst_*	1sft	1.34 (44%)	1.25 (44%)	0.71 (48%)	0.48 (70%)
Alr*_Bax_*	3ha1	1.54 (43%)	1.47 (43%)	0.75 (46%)	0.53 (68%)
Alr*_Mtb_*	1xfc	1.67 (33%)	1.62 (32%)	1.13 (36%)	0.72 (51%)
DadX*_Pao_*	1rcq	2.14 (30%)	1.60 (29%)	1.36 (32%)	0.82 (47%)

† Calculated using monomer *A*.

‡ Calculated using residues 2–241.

§ Calculated using residues 242–382.

¶ Calculated using residues 37–43, 61–65, 82–86, 101–105, 127–140, 163–171, 198–205, 216–223 and 351–358 from monomer *A* and residues 263–266, 309–314 and 283–287 from monomer *B*.

**Table 3:** Kinetic parameters for the racemization between L-alanine and D-alanine by alanine racemases NR: value not reported.

	L→D direction		D→L direction		
Alanine racemase	*K*_m_ (m*M*)	*V*_max_ (U mg^−1^†)	*K*_m_ (m*M*)	*V*_max_ (U mg^−1^†)	*K*_eq_‡
Alr*_Sas_*§	2.77	250	0.89	91	0.89
Alr*_Gst_*[Table-fn tfn10]	4.25	2550	2.67	1400	1.14
Alr*_Bax_*[Table-fn tfn11]	2.8	101	NR	NR	NR
Alr*_Mtb_*[Table-fn tfn12]	1.2	0.51	1.1	0.46	1.02
DadX*_Pao_*†	1.40	155	1.40	134	1.16

† One unit is defined as the amount of enzyme which catalyzes the racemization of 1 µmol of substrate per minute.

‡ *K*_eq_ = *V*_max(L→D)_ × *K*_m(D→L)_/*V*_max(D→L)_ × *K*_m(L→D)_.

§ Current work; assay performed at 303 K.

¶ Inagaki *et al.* (1986 ▶); assay performed at 310 K.

†† Couñago *et al.* (2009 ▶); assay performed at 296 K.

‡‡ Strych *et al.* (2001 ▶); assay performed at 296 K.

§§ Strych *et al.* (2000 ▶); assay performed at 296 K.
